# Function of a novel plakophilin-2 mutation in the abnormal expression of connexin43 in a patient with arrhythmogenic right ventricular cardiomyopathy

**DOI:** 10.3892/etm.2014.2145

**Published:** 2014-12-18

**Authors:** PEI-NING WANG, SHU-LIN WU, BIN ZHANG, QIU-XIONG LIN, ZHI-XIN SHAN

**Affiliations:** Department of Cardiology, Guangdong Cardiovascular Institute, Guangdong Academy of Medical Science, Guangdong General Hospital, Guangzhou, Guangdong 510100, P.R. China

**Keywords:** arrhythmogenic right ventricular cardiomyopathy, desmosome, connexin43, gap junction, plakophilin-2 gene

## Abstract

Arrhythmogenic right ventricular cardiomyopathy (ARVC) is a desmosomal disease. Desmosomes and gap junctions are important structural components of cardiac intercalated discs. The proteins plakophilin-2 (PKP-2) and connexin43 (Cx43) are components of desmosomes and gap junctions, respectively. This study was conducted to determine whether Cx43 expression is affected by the mutation of the PKP-2 gene in patients with ARVC. A novel mutation was detected in a typical patient with ARVC. The mutated gene was transfected into rat mesenchymal stem cells expressing Cx43 through a pReversied-M-29 plasmid. Cx43 expression was detected using quantitative polymerase chain reaction analysis. Cx43 expression was significantly decreased in the mutant PKP-2 group compared with that in the wild-type PKP-2 group. In conclusion, PKP-2 affected Cx43 expression at the gene transcription level in the patient with ARVC.

## Introduction

Arrhythmogenic right ventricular cardiomyopathy (ARVC) is a familial disease that is characterized by right ventricular fibrofatty degeneration, which promotes right ventricular dysfunction and arrhythmogenesis (1–3). In a previous study, we reported the electrocardiogram (ECG) features of ARVC in China (4). Given that mutations in desmosomal proteins, including desmin, desmoplakin, desmoglein, desmocollin, plakoglobin and plakophilin-2 (PKP-2), are important in the pathogenesis of ARVC, this condition has been termed a desmosomal disease (5–7).

Cardiomyocytes are held together at so-called intercalated discs, which are composed of gap junctions, fascia adherens junctions and desmosomes. Gap junctions are the primary structures that underlie the electrical conduction between myocytes. Gap junctions consist of connexons, which are delicate interdigitating structures composed of hexameric assemblies of connexins from cells on both sides of each junction (8–10). The mechanical stability of the gap junction is provided by the more rigid structure of adherens junctions and desmosomes co-localized in the intercalated disc region. PKP-2 is an important desmosomal protein that interacts with plakoglobin (11). In a previous study, we detected a mutation in the PKP-2 gene in a Chinese patient with ARVC (12).

To evaluate the effect of mechanical coupling on electrical coupling in ARVC, Oxford *et al* (13) demonstrated that the inhibition of PKP-2 expression resulted in reduced expression and abnormal subcellular localization of connexin43 (Cx43), a typical gap junction protein. Further to this, two questions were proposed: i) Whether the expression of ARVC-related PKP-2 mutants or the silencing of the wild-type protein led to the redistribution of Cx43, and ii) whether the decrease in Cx43 level was attributable to changes in gene transcription. The aim of the present study was to answer these two questions. A novel mutation was detected in a typical patient with ARVC from our hospital (Guangdong General Hospital, Guangzhou, China). The mutation was used to study the association between PKP-2 and Cx43.

## Subjects and methods

### Clinical data

A 62-year-old male was referred to the hospital due to recurrent faintness. The patient’s resting ECG showed T-wave inversion in the V_1-4_ leads. Sustained ventricular tachycardia of the left bundle branch block morphology with an inferior axis was recorded during the inpatient period, and signal-averaged ECG recordings showed positive late potentials. Echocardiography revealed an enlarged, hypokinetic right ventricle with a paper-thin free wall. Following a diagnosis of ARVC, the patient provided informed consent for genetic analysis. The patient received radiofrequency current catheter ablation four times, but the procedures were unsuccessful. He was then instead given an implantable cardioverter defibrillator (Medtronic, Minneapolis, MN, USA). This study was conducted in accordance with the Declaration of Helsinki and with approval from the Ethics Committee of Guangdong General Hospital. Written informed consent was obtained from the participant.

### Genetic analysis

The genomic DNA and RNA of the patient were extracted from peripheral blood cells using standard methods. All 14 exonic and adjacent intronic sequences of the PKP-2 gene were examined using polymerase chain reaction (PCR) amplification combined with direct sequencing. The primers are listed in Table I (Promega Biotechnology Ltd., Shanghai, China). The cDNA of the patient was obtained from the mRNA by reverse transcription PCR (RT-PCR). RT-PCR combined with direct sequencing was used to detect the mutation. The primers are listed in Table II (Promega Biotechnology Ltd.).

### Construction of the plasmids

The cDNA for the wild-type and mutant PKP-2 was obtained in our laboratory by RT-PCR using the mRNA derived from the peripheral blood cells of normal controls and from the patient with ARVC. There were 30 normal, healthy controls, all of which provided informed consent. The primers were as follows: Forward, 5′-GAAGGAATTCGG TACATGGCAGCCCCCGGCGCCCCAGC-3′ and reverse, 5′-TCGCGATCGCCCGGGCTCTTCTAGTCT TTAAGGGAGTGGTAGGCTT-3′. The cDNA clone nucleotides of the wild-type and mutant PKP-2 were inserted in a pReversied-M-29 vector (Promega Biological Products Ltd., Shanghai, China) carrying green fluorescent protein by digestion with the enzymes *Eco*RI and *Asi*SI, respectively. The nucleotides were then verified by sequencing.

### Cell culture and transfections

Rat mesenchymal stem cell (rMSC) lines expressing Cx43 were provided by Dr Xiaohong Li (Medical Research Center, Guangdong Cardiovascular Institute, Guangdong General Hospital, Guangzhou, China). The cell lines were maintained at 37°C in a humidified atmosphere of 5% CO_2_ and 95% air. The cell lines were cultured in Dulbecco’s Minimal Essential Medium with penicillin/streptomycin and 10% fetal bovine serum (Promega Biotechnology Ltd.). The wild-type and mutant PKP-2 plasmids were transfected into rMSC lines using Lipofectamine^®^ 2000 reagent (Invitrogen Life Technologies, Carlsbad, CA, USA) according to the manufacturer’s protocol. rMSC RNA and protein were extracted 48 h after transfection.

### PKP-2 and Cx43 expression

mRNA was purified and quantified from the rMSC lines 48 h after transfection. The expression of PKP-2 and Cx43 was observed at the transcriptional level using RT-PCR and SYBR green dye (Promega Biotechnology Ltd.). The primers used were as follows: i) PKP-2 (120 bp) forward, 5′-GCTGCTTCCGTCCTTCTGTA-3′ and reverse, 5′-GGAGTGGTAGGCTTTGGCA-3′; Cx43 (111 bp) forward, 5′-AGAATGTAGCAGTAATCGCCA-3′ and reverse, 5′-TGA CTAAAGCCCGTTTACAGTAC-3′; β-actin (155bp) (internal reference) forward, 5′-TGTTGCCCTAGACTTCGAGCA-3′ and reverse, 5′-GGACCCAGGAAGGAAGGCT-3′. A mixture of all reagents was prepared as follows: 10 μl SYBR green reaction buffer, 7 μl sterile purified water, 1 μl 20 μM forward primer, 1 μl 20 μM reverse primer and l μl purified mRNA (≤250 ng/reaction). Samples were run in triplicate on a 7300 Real-Time PCR System in 96-well MicroAmp^®^ optical plates (both Applied Biosystems, Foster City, CA, USA). RT was performed at 50°C for 30 min, followed by inactivation of RT at 95°C for 15 min, and 40 cycles of 95°C (30 sec), 54°C (30 sec), and 72°C (40 sec). Relative quantities were calculated using Stratagene instrument software (Agilent Technologies, Santa Clara, CA, USA).

### Statistical analysis

Differences in mRNA content among the control, wild-type and mutant groups were evaluated using two-way analysis of variance, with α of ≤0.05 considered significant. All results are expressed as the mean ± standard error.

## Results

### Genetic analysis

**Figure 1:**
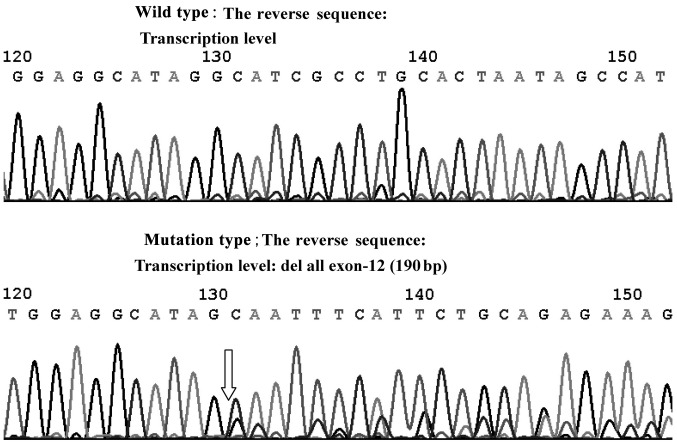
Deletion mutation of plakophilin-2 at the RNA level.

The junction of intron-10 and exon-11 in PKP-2 showed the deletion mutation (2 bp) (c.2146-1-2146 Del GA) at the DNA level by direct sequencing. Direct sequencing also revealed the deletion of exon-12 (190 bp) at the transcriptional level (Fig. 1).

### PKP-2 expression

**Figure 2:**
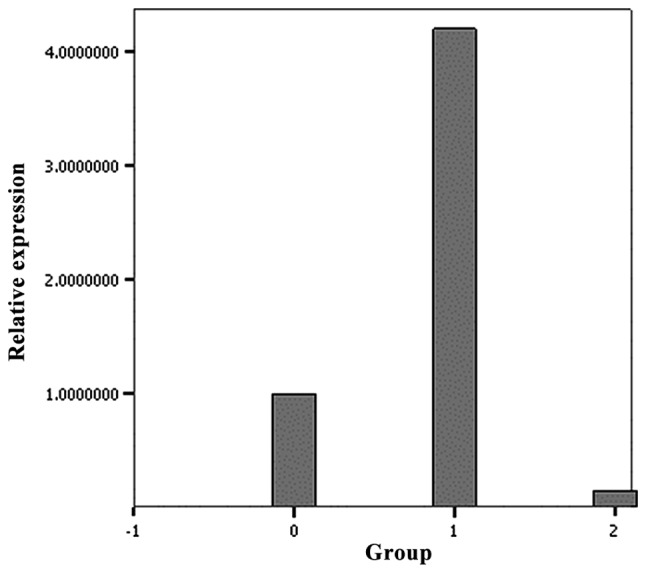
Plakophilin-2 mRNA expression among the different groups. 0, control group; 1, wild-type group; 2, mutant-type group.

RT-PCR was used to detect the differential expression of PKP-2 mRNA. The level of mRNA expression observed in the mutant PKP-2 group was significantly lower than that in the wild-type group (Fig. 2).

### Cx43 expression

**Figure 3:**
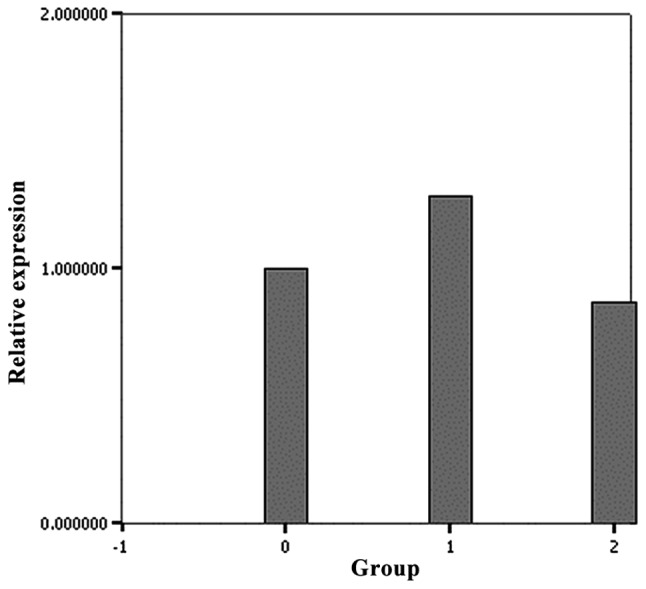
Connexin43 mRNA expression among the different groups. 0, control group; 1, wild-type group; 2, mutant-type group.

RT-PCR of the mutant PKP-2 group showed a significantly lower level of Cx43 mRNA compared with the wild-type PKP-2 (Fig. 3). The mRNA expression of Cx43 in the wild-type group and control were not significantly different (P=0.085).

## Discussion

The main clinical feature of ARVC is ventricular tachycardia, which originates from the right ventricle and can lead to syncope or sudden cardiac death (14–16). Ventricular arrhythmia is attributed to disordered electrical signals; however, the factor inducing the gap junction to affect the conduction of electrical signals remains unknown. We propose that one of the mechanisms is the effect of PKP-2 mutation on the decrease in the expression of Cx43, an important part of the gap junction.

Cx43 expression has also been found to be significantly reduced in patients with Naxos disease. Naxos disease is characterized by ARVC, palmoplantar keratoderma and wooly hair, which suggests that the integrity of the desmosome is a prerequisite for the normal functions of the gap junction (17,18).

In the present study, a novel PKP-2 mutation was detected in a patient with ARVC; this mutation not only appeared at the DNA and RNA levels, but also induced changes in the amino acid sequence (Table III). The changes to the base pairs and amino acids occurred when exon-12 was spliced. The amino acid sequence of protein number 767 changed from GCC to GCU; however, the protein remained as alanine. The next protein along, protein number 768, changed from lysine to methionine, due to the amino acid sequence change from AAA to AUG. In answer to the questions raised by Oxford *et al* (13), it was found that the expression of ARVC-related PKP-2 mutations could lead to a similar redistribution of Cx43. In addition, it was demonstrated that the decrease in Cx43 content was attributable to changes observed at the level of gene transcription.

In a previous study, Fidler *et al* (19) performed endomyocardial biopsies of 27 patients with ARVC with mutated PKP-2 genes, as confirmed by sequencing. The biopsied tissues were then assessed by immunofluorescence to visualize intercalated disc proteins. Reduced Cx43 expression and localization to the intercalated disc were observed in heterozygous human PKP-2 volunteers, which potentially explains the delayed conduction and propensity to develop arrhythmia in this disease.

The mechanisms by which mutations in mechanical junctions affect the rhythm of the heart remain unknown (8). The results of the present study indicate that mutations in PKP-2, an important structural protein that stabilizes cardiac gap junctions, contribute to the pathophysiology underlying AVRC. It is believed that the effect of mutant PKP-2 may be a consequence of alterations in the electrical conductions in the heart. The potential effect of PKP-2 mutations on mitochondrial function and myocyte apoptosis merits further study.

In conclusion, this study is the first to investigate the pathophysiological processes of ARVC using the PKP-2 mutation, which was screened in a clinical setting, to determine whether the expression of Cx43 has a function in the development of ARVC. In future studies we aim to collect more data from patients with ARVC and detect the same mutation to further reveal the nature of this disease.

## Figures and Tables

**Table I:** Primers used to amplify the exons of plakophilin-2.

Exon (protein no.)	Forward (5′-3′)	Reverse (5′-3′)
Pkp-2-1 (544)	GCCCACGAGGCCGAGCTCC	AGCAAGTCGGTCATACCGAAGA
Pkp-2-2 (425)	TACTTGTTCTTGGCCTTCATTAC	GCACTAGGATGTAAGAATGTTTC
Pkp-2-3 (920)	TTCAGAGAAACGGACATGTTGG	AAGGGCTTCCAGAGATAAGTGA
Pkp-2-4 (302)	TAGGCAGGAGGAGGGAGGT	CCAAAGTGCTGGGAATAT
Pkp-2-5 (479)	CAAGAGCCTCAGTTGTGCTAC	CCTTCTCTAGCATAACAATGAG
Pkp-2-6 (417)	TAACTATACAGGCTCTTATTTCAG	CTGGAGTGTAGTGGCACAATC
Pkp-2-7 (363)	CATAGCCCTGGAGTTGATGG	AAGAACCAAAGGCAGAATATATCC
Pkp-2-8 (283)	ACAAAGACCTGTTGGATACACA	CTCAGTAAATGAATCAGTGAATAA
Pkp-2-9 (431)	TTCTAGCCATACTCATTGCATTTC	ACTTGGTATATATCGGCACTATT
Pkp-2-10 (481)	TTCTATTTCAAGGGCTTCTTATG	AGCCTGACTTGACTTTGCATAA
Pkp-2-11 (339)	TCAACCTCTGGTAATCTACAGA	CATTGCATTGTATCTTCAGCATG
Pkp-2-12 (479)	AGTGAGCCAAGATGGTGCCA	CAGCAAACAGGATGTAAAGCC
Pkp-2-13 (358)	GGCCTGACTTCATGGATGGCT	CCTTTCACGTTTCTGTTTGCTTA
Pkp-2-14 (589)	CTGGGAAGAAATCGCTAAAA	GCAGAACAATACACTGGAGGC

**Table II:** Primers used for the reverse transcription polymerase chain reaction.

No.	Forward (5′-3′)	Reverse (5′-3′)
1	ATGGCAGCCCCCGGCGCCCC	GCCTGGCCGACAGTCAAGTG
2	GTGGATTCCAGCGGGAGGAG	GAGAGGTTATGAAGAATGCACACA
3	ACCATTGCAGATTACCAGCCAGA	TCAGTCTTTAAGGGAGTGGTAGG

**Table III:** Changes in the base pairs and amino acids when exon-12 is spliced.

WT base	WT amino acid	MT base	MT amino acid	Protein number
AAU	N	AAU	N	764
GAA	E	GAA	E	765
AUU	I	AUU	I	766
GCC	A	GCU	A	A767A
AAA	K	AUG	M	K768M
GAA	E	CCU	P	E769P
ACU	T	CCA	P	T770P
CUC	L	ACA	T	L771T
CCU	P	AAG	K	P772K

A, alanine; K, lysine; M, methionine; E, glutamic acid; P, proline; T, threonine; L, leucine; N, asparagine; I, isoleucine; WT, wild-type; MT, mutant-type.
